# Design of Large‐Aperture Hybrid Refractive Metalens System

**DOI:** 10.1002/nap2.70301

**Published:** 2026-09-18

**Authors:** Yinpeng Ma, Huachao Cheng, Shijie Wei, Ben Xue, Xihang Yang, Tengfei Wu, Yue Wang, Ruixiang Yang, Qingqing Guo, Teli Xi

**Affiliations:** ^1^ Xi'an Key Laboratory of Computational Imaging, School of Optoelectronic Engineering Xidian University Xi'an Shaanxi China; ^2^ Xi'an Institute of Optics and Precision Mechanics Chinese Academy of Sciences Xi'an Shaanxi China

**Keywords:** computational imaging, hybrid refractive metalens, large‐scale simulation, LWIR

## Abstract

Metalenses flexibly control wavefronts in compact systems, but large‐aperture broadband imaging remains challenging because phase compensation increases rapidly and full‐wave simulations are computationally prohibitive. We propose a wavefront‐constrained joint optimization framework for large‐aperture hybrid refractive metalens imaging systems. The method couples ray tracing of the refractive lens, angular spectrum propagation of the metalens‐modulated field, and manufacturable meta‐atom phase mapping, enabling system‐level optimization of the hybrid optical path without full‐aperture full‐wave modeling. The refractive lens provides the primary focusing power; the metalens introduces an optimized continuous phase profile to compensate residual aberrations and reduce broadband image‐plane wavefront errors. Here, we design and fabricate a 40‐mm‐aperture, F/2 hybrid refractive metalens system operating over the 7.7–9.5 μm long‐wave infrared (LWIR) band. Compared with the single refractive lens baseline, the optimized hybrid design reduces the root‐mean‐square (RMS) wavefront error and peak‐to‐valley (PV) value by 44% and 37%. The fabricated system achieves modulation transfer function (MTF) values higher than 0.2 at the detector Nyquist frequency. A frequency‐aware neural reconstruction module suppresses residual blur and noise. These results demonstrate that the method provides a practical route to scaling refractive–metalens optics toward large‐aperture, compact, and broadband infrared imaging systems.

## Graphical abstract

An all‐germanium hybrid refractive–metalens system was constructed by integrating a planar Ge metalens with a single aspheric Ge lens, enabling compact wavefront correction in the LWIR band. The all‐Ge hybrid architecture simplifies conventional multi‐element infrared optics into a lightweight integrated imaging system while maintaining effective broadband imaging performance.

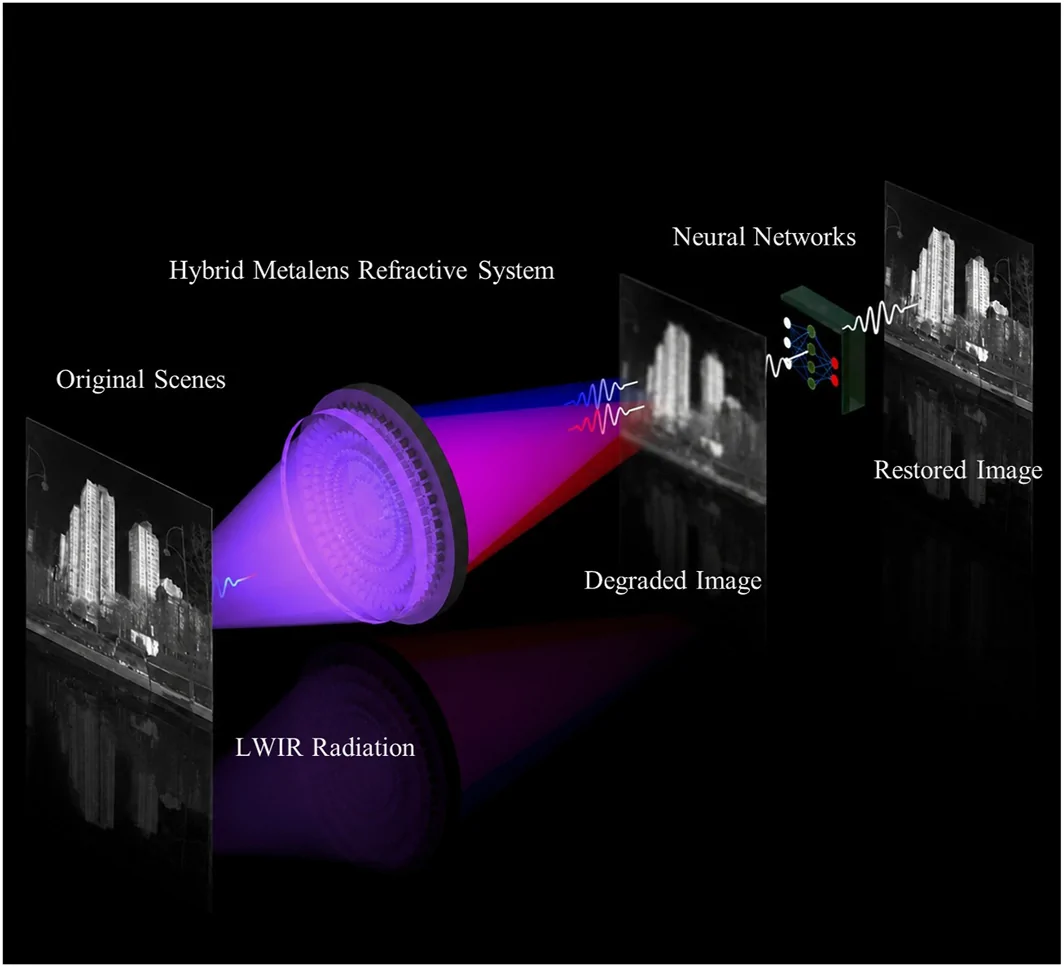

## 1 Introduction

Infrared imaging technology enables stable passive target detection in nighttime and complex environments, with all‐weather operational capability [1, 2, 3]. Its passive imaging mechanism has led to extensive research and application in fields such as nighttime surveillance [4], remote sensing [5], biomedical imaging [6], and vegetation monitoring [7]. Existing infrared imaging systems rely on multi‐element lens assemblies, complex optical‐mechanical structures, and high‐cost infrared materials. This results in bulky and heavy systems with elevated manufacturing and integration costs, making it challenging to meet the lightweight and low‐cost requirements of airborne, satellite‐based, and portable platforms.

Simplifying infrared imaging systems is essential for compact and lightweight optical architectures. Aspheric and diffractive optical elements have been widely used to reduce lens count and optical track length. Metasurfaces have recently emerged as a promising route for further optical system simplification. Composed of two‐dimensional planar arrays featuring subwavelength‐scale structures, metasurfaces offer ultra‐thin and lightweight advantages while enabling flexible control over light's phase [8, 9, 10], amplitude [11, 12], and polarization [13, 14] characteristics. They demonstrate significant potential for achieving complex functionalities within optical systems. However, the inherent wavelength‐dependent modulation characteristics of metalenses exacerbate dispersion issues [15] and pose significant design and fabrication challenges when scaling to large apertures [16, 17]. Hybrid refractive metalens optical systems combine metalens with traditional refractive elements [18, 19, 20], enhancing dispersion correction capabilities while reducing system weight. These capabilities make metasurfaces promising for implementing complex optical functionalities in compact imaging systems. Previous studies have demonstrated that introducing a metalens as a wavefront modulation unit behind a refractive lens enables broadband or achromatic imaging within a certain range while maintaining system compactness, validating the hybrid architecture's feasibility for dispersion compensation [21]. Further work has extended metamaterials into system‐level aberration correction, utilizing them as auxiliary correction elements to enhance imaging performance along the optical axis or within partial fields of view [22]. Researchers have also employed inverse design methods to holistically optimize the phase distribution of metamaterials within hybrid systems, achieving aberration suppression and imaging validation across a large field of view in the LWIR spectrum [23]. Although the above work has made progress in dispersion control and system compactness, the effective aperture of existing folded hybrid systems remains relatively small due to constraints on the phase modulation range and efficiency of metamaterial units. Overall imaging quality still lags significantly behind traditional optical systems.

Post‐imaging algorithmic restoration serves as a crucial complementary approach beyond optical design. Neural network algorithms demonstrate superior nonlinear representation capabilities in denoising [24, 25, 26] and super‐resolution [27, 28, 29, 30] visual tasks, enabling more effective detail recovery and artifact suppression. Most current neural network‐based infrared image enhancement methods focus on pixel‐level processing, simplifying the imaging process into an end‐to‐end mapping from degraded to clear images. They do not explicitly model the optical transfer characteristics of the imaging system and therefore show limited generalization to real‐world system degradations. To overcome this limitation, computational imaging techniques have emerged [31, 32, 33, 34]. Computational imaging goes beyond merely optimizing the image itself. By tightly integrating computation with the imaging process, it relaxes the constraints of traditional optical imaging. This approach introduces system priors into the reconstruction process and compensates for imaging degradation, providing a more physically coherent framework.

In this work, we propose a joint optimization method for large‐aperture LWIR hybrid refractive metalens imaging systems. The method uses ray tracing to calculate the wavefront transmitted by the aspheric Ge lens and employs diffraction propagation to model the light‐field modulation introduced by the metalens. The optimized compensation phase is subsequently mapped to practical metalens structures for device implementation. Based on the optimized design, an all‐germanium hybrid prototype is fabricated and experimentally characterized over the 7.7–9.5 μm band. Compared with the single refractive lens baseline, the hybrid design reduces the RMS wavefront error and PV value by approximately 44% and 37%, respectively, and achieves measured MTF values higher than 0.2 at the detector Nyquist frequency. With post‐imaging neural reconstruction, the residual blur and noise are further suppressed, improving the visual quality of real infrared images. These results demonstrate that the proposed joint optimization method provides a feasible route for compact, lightweight, and broadband LWIR imaging systems, with potential applications in airborne, spaceborne, and portable infrared imaging platforms.

## 2 Method and Design

### 2.1 Optimized Design of Hybrid Refractive Metalens System

We developed an optical–computational design framework for the large‐aperture hybrid refractive metalens imaging system, as shown in Figure 1. In this framework, the optical system is first optimized to suppress the dominant wavefront errors and reduce the physical blur introduced by aberrations, whereas the neural network is used as a back‐end reconstruction module to compensate for the remaining image degradation caused by residual blur, detector noise, fabrication errors, and assembly imperfections. The optical design is implemented through a two‐level optimization strategy. In the first level, a forward wavefront‐constrained genetic algorithm is used to optimize the curvature, thickness, conic constant, aspheric coefficients, and axial spacing of the refractive lens, together with the continuous phase distribution of the metalens. For each candidate design, ray tracing is used to calculate the wavefront transmitted by the Ge aspheric lens, and the angular spectrum method (ASM) is then used to propagate the field through the metalens to the image plane. The fitness of each candidate is evaluated from the image‐plane wavefront error and imaging response of the complete hybrid optical path. In the second level, the optimized continuous phase is mapped to practical Ge meta‐atoms using a multi‐wavelength unit‐cell library. The system‐level refractive metalens wavefront optimization and the meta‐atom structure realization are performed as two sequential stages, whereas the neural network further restores the residual degradation that cannot be fully removed by optical design alone.

**FIGURE 1:**
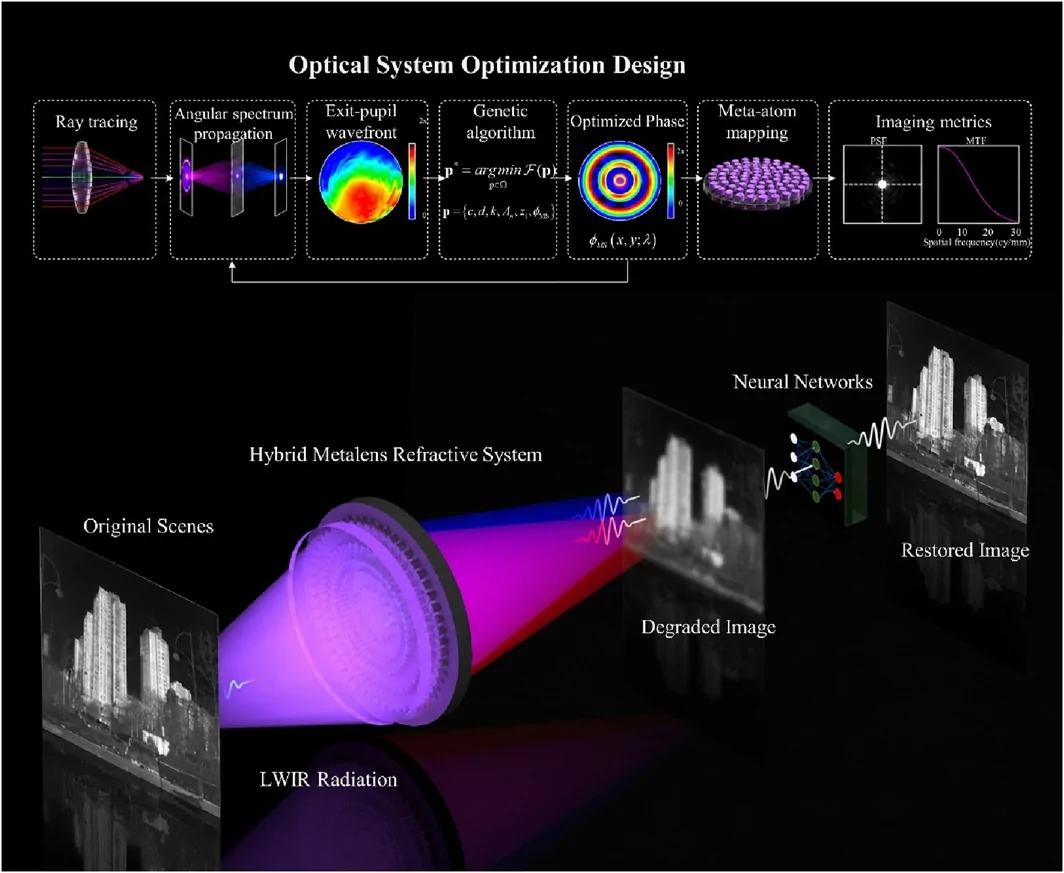
Schematic diagram of a computational imaging system based on a hybrid refractive metalens optical system.

Cooled infrared imaging systems usually require five or more refractive lenses to achieve high image quality, which increases the optical track length, assembly complexity, and fabrication cost [35]. To simplify the optical architecture, we use a single aspheric refractive lens combined with a planar metalens, as shown in Figure 2a. The aspheric lens provides the main focusing power and controls the dominant geometrical aberrations, whereas the metalens introduces an additional wavelength‐dependent phase modulation *ϕ*_MS_(*x*_1_, *y*_1_; *λ*). In this configuration, the imaging function is not realized by increasing the number of refractive elements, but by redistributing the correction capability between the aspheric surface and the planar phase modulation layer.

**FIGURE 2:**
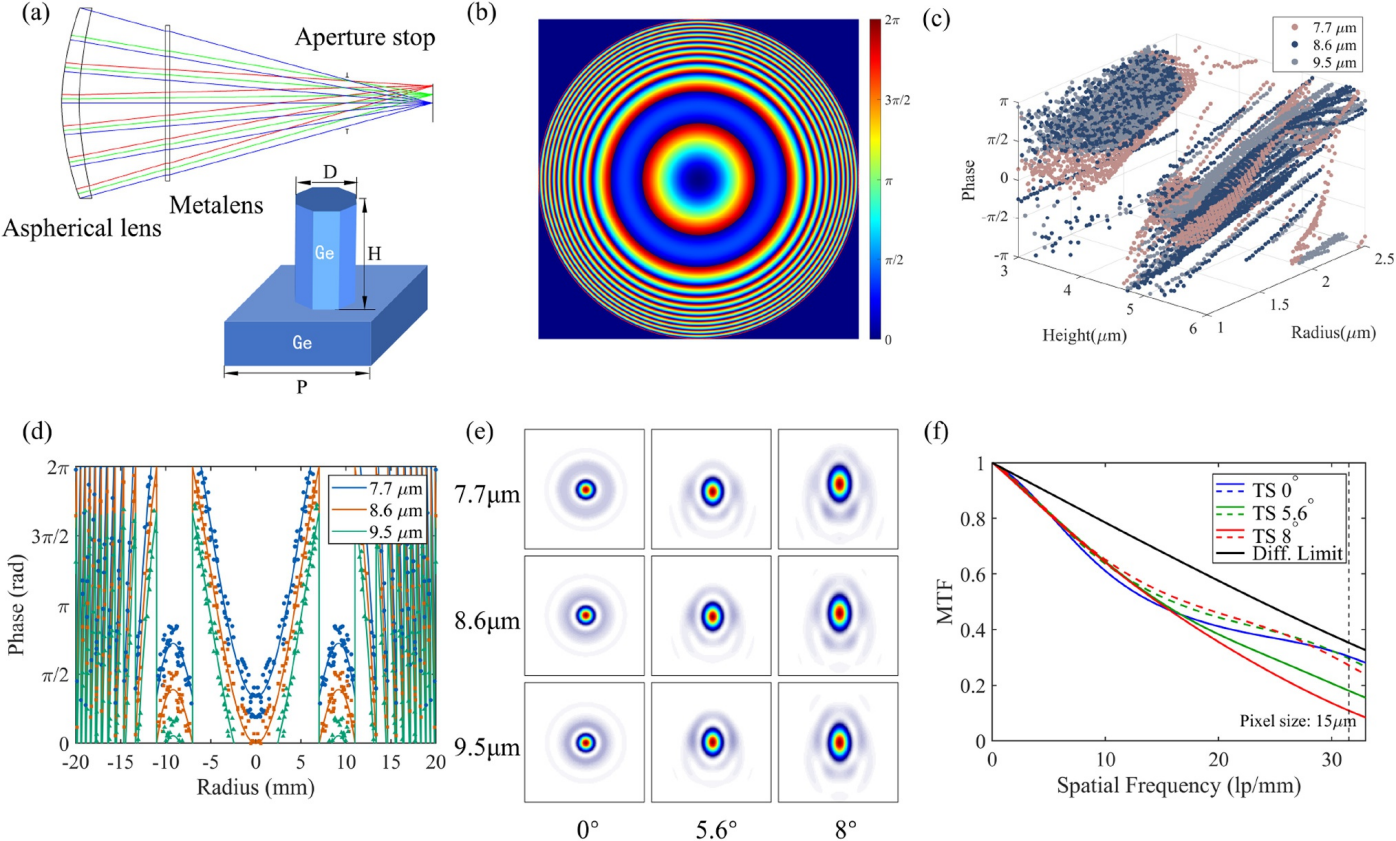
Optimization results of the hybrid refractive metalens system. (a) Optical layout of the hybrid refractive metalens system. (b) Optimized metalens phase. (c) Metalens unit‐cell library. (d) Ideal target phase and selected practical phase profiles. (e) Optimized PSF at different wavelengths and field angles. (f) The simulation results of the MTF.

For infrared imaging based on large‐aperture metalens, when the aperture is much larger than the wavelength, performing a full‐wave simulation across the entire aperture becomes impractical. When D/*λ* > 1000, a full‐wave model with 10 sampling points per wavelength would require more than (10D/*λ*)^2^ > 10^8^ lateral grid points for a single cross‐section alone. In our system, the aperture‐to‐wavelength ratio reaches D/*λ* ≈ 4.65 × 10^3^, which means that, without considering propagation depth, material interfaces, and time steps, the number of lateral sampling points alone exceeds 2.1 × 10^9^. As the device area increases, full‐structure finite‐difference time‐domain (FDTD) simulations become computationally prohibitive. Therefore, we adopted a forward wavefront modeling approach. By performing ray tracing calculations for the refractive lens, we obtained the ray directions, optical path lengths, and wavefront distributions after passing through the aspheric surface. The aspheric surface shape is represented as follows:

(1) $$Z\left(x,y\right)=\frac{c\left(x^{2}+y^{2}\right)}{1+\sqrt{1-\left(1+k\right)c^{2}\left(x^{2}+y^{2}\right)}}+\sum_{i=2}^{n}A_{i}{\left(x^{2}+y^{2}\right)}^{i},$$

where *Z*(*x*,*y*) represents the surface elevation, *c* denotes the vertex curvature of the refractive lens surface, *k* is the conic constant, and *A*_*i*_ denotes the higher‐order aspheric coefficients. This surface profile function determines the propagation path of light rays within the refractive lens and their cumulative optical path length, thereby influencing the system's initial wavefront distribution.

Through ray tracing calculations, we obtain the distribution of optical path differences at the image‐side exit pupil of the refractive lens, represented as the image‐side exit pupil wavefront *W*_lens_(*u*,*v*,*λ*). This wavefront serves as the starting point for modeling the hybrid system as a wavefront, characterizing the imaging properties of the refractive optical section under given structural parameters.

The ASM propagates the complex field from the refractive lens exit pupil through the metalens to the image plane. This wave‐optics treatment captures the wavelength‐dependent phase modulation, diffraction, and interference that govern the image‐plane response of each candidate metalens phase profile. For each candidate optical configuration generated by the genetic algorithm, ray tracing was performed to calculate the optical path length and the image‐side wavefront *W*_lens_(*x*,*y*), transmitted by the Ge aspheric lens. The corresponding complex field was expressed as follows:

(2) $$U_{\text{lens}}\left(x,y\right)=A_{\text{lens}}\left(x,y\right)\exp\left[i\cdot 2\pi /\lambda \cdot W_{\text{lens}}\left(x,y\right)\right],$$

where *A*_lens_(*x*,*y*) denotes the amplitude distribution at the lens exit pupil, *λ* is the operating wavelength, and 2π/*λ* represents the free‐space wavenumber. The field *U*_lens_ was then propagated to the metalens plane using ASM, where the candidate phase *ϕ*_MS_(*x*_1_,*y*_1_;*λ*) was applied as a thin phase modulation layer. The modulated field was further propagated to the image plane to obtain the final wavefront *W*_img_. The wavefront error, point spread function (PSF), and MTF derived from the image‐plane field were used as the fitness evaluation for each candidate hybrid configuration. This fitness evaluation was used to guide the first‐level genetic optimization of the system parameters, including the refractive curvature *c*, lens thickness *d*, conic constant *k*, aspheric coefficients *A*_2_, *A*_4_, and *A*_6_, axial spacing *z*_1_, and the continuous metalens phase *ϕ*_MS_. Each candidate represents a complete hybrid design comprising refractive lens parameters and metalens phase profile. Candidates with lower fitness values are retained through selection, crossover, and mutation, and the best candidate after convergence is selected for the subsequent meta‐atom mapping. The detailed genetic operations, including population initialization, selection, crossover, mutation, and convergence criteria, are provided in Supporting Information S1: Note S1.

Germanium was selected as the material for the optical components because of its high transmittance in the LWIR band and the availability of mature fabrication processes in the field of infrared optics. In the first‐level wavefront‐constrained genetic optimization, the thickness and curvature of the germanium lens were constrained jointly to balance optical performance and manufacturability. The lens thickness was searched within the range of 2.0–5.0 mm, and the optimized value was *d* = 4.0 mm. The aspheric coefficients were selected by balancing aberration correction and fabrication tolerance. The conic constant was constrained within −1 ≤ *k* ≤ 0, and only the *A*_2_, *A*_4_, and *A*_6_ higher‐order aspheric terms were retained. The maximum surface sag variation was limited to 0.35 mm, and local surface slopes were restricted to < 8° to avoid excessively steep edge profiles. Because higher‐order terms such as *A*_8_ contributed less than 1% to the reduction of the root‐mean‐square wavefront error but increased local surface slope and alignment sensitivity, the aspheric expansion was limited to *A*_6_.

The realized discrete phase was reintroduced into the angular spectrum propagation model for the final image‐plane evaluation. Based on these optimization constraints, the final refractive configuration was determined, and the residual wavefront was further converted into the required metalens compensation phase. The optimized compensation phase at the central wavelength of 8.6 μm is shown in Figure 2b, exhibiting a continuous radial phase variation across the aperture. We mapped the continuous compensation phase to Ge unit‐cell geometries at successive radial positions. At each position, the prescribed physical constraints guided the selection of a suitable geometry from a multi‐wavelength library. The unit cell was designed as a regular octagonal prism to approximate rotational symmetry while maintaining straight sidewalls for fabrication. As shown in Figure 2c, the height *H* was swept within 3–6 μm, and the radius *r* was swept within 1–2.5 μm. The optimized global parameters were obtained as *H* = 4.5 μm, providing full 0–2π phase coverage with an average transmittance above 60%. Considering the fabrication resolution, inter‐element spacing, and the requirement for sufficient phase coverage, the lattice period *P* was selected as 4 μm for the metalens design.

After joint optimization, the system's image‐plane wavefront distribution showed significant improvement. By simultaneously adjusting the curvature, thickness, and a sphericity coefficient of the refractive lens, while introducing metalens phase to compensate and reshape the propagating wavefront, overall undulations in the image‐plane wavefront were markedly suppressed. As shown in Supporting Information S1: Figure S1, compared with the pre‐optimization state, wavefront errors decreased substantially across the entire aperture range, with the wavefront distribution approaching the target reference wavefront. This improvement remained consistent across different field‐of‐view (FoV) positions. Supporting Information S1: Table S1 shows that the hybrid refractive metalens system reduces the RMS wavefront error by approximately 44% and the PV value by about 37% compared with the single refractive lens. As shown in Figure 2d, the phase response of the selected Ge octagonal unit cells agrees well with the ideal target phase at the center wavelengths, indicating that the optimized structure set can accurately reproduce the required compensation phase while maintaining broadband phase‐matching capability. The simulated PSF is shown in Figure 2e. The results demonstrate the performance of the optical system across different wavelengths and FoVs. The PSF remains relatively uniform within the 0°–8° range, whereas off‐axis aberrations appear at larger FoV. The corresponding MTF results, shown in Figure 2f, validate the high imaging quality. Despite the presence of off‐axis distortions, the MTF remains consistently above 0.2 at 33 lp/mm across the entire spectral range of 7.7–9.5 μm.

### 2.2 Optical Imaging Simulation Model

To accurately simulate the imaging process of the hybrid refractive metalens system, we derive the PSF based on the continuous wavefront on the optimized exit pupil plane. This PSF is then convolved with the target intensity distribution to obtain the degraded image, followed by noise addition to simulate real imaging conditions. Because of wavefront variations, the PSF becomes a spatially varying function, exhibiting distinct characteristics across different regions of the imaging plane. To account for this spatial variation, we partition the entire imaging plane into multiple smaller regions and compute the PSF for each region separately. The final imaging result is obtained by superimposing the contributions from all regions and can be expressed as follows:

(3) $$I_{\deg,r}\left(x,y\right)=\iint I_{\text{obj},r}\left(x^{\prime },y^{\prime }\right)\cdot \left|h_{r}\left(x-x^{\prime },y-y^{\prime }\right)\right|dx^{\prime }dy^{\prime }+N.$$

The PSF for each region *r* can be expressed as *h*_*r*_(*x*,*y*), where (*x*,*y*) denotes the coordinates within that region. The target intensity distribution within each region is represented by *I*_obj,*r*_($x^{\prime },y^{\prime }$). By convolving the target intensity with its corresponding PSF, we obtain the degraded image for each region. To form a complete degraded image across the entire imaging plane, we stack the degraded images from all regions. To accurately simulate real imaging environments and evaluate the impact of noise on imaging quality, we introduced detector thermal noise into the simulation, as depicted in Supporting Information S1: Figure S2.

### 2.3 Neural Network Algorithms for Image Process Enhancement

Residual aberrations remaining after hybrid refractive metalens optical design are corrected by a neural network reconstruction algorithm. We selected diffusion models as the core method, which predict noise by training a denoising autoencoder to estimate the inverse process of a Markov diffusion process. This approach demonstrates remarkable stability and controllability in image generation and enhancement tasks. However, existing diffusion models predominantly perform pixel‐level processing in the spatial domain, failing to fully leverage frequency domain information. We propose a diffusion model enhancement method based on frequency domain processing to overcome the limitations of traditional spatial domain processing and further enhance imaging quality. This improvement enables more effective utilization of frequency domain features, thereby addressing the shortcomings of existing methods in complex imaging scenarios.

The network architecture of our proposed Infrared frequency‐enhanced diffusion network (IFEDiff‐Net), as shown in Figure 3a, adopts a wavelet‐based dual‐branch encoder–decoder architecture with frequency‐aware feature fusion. This architecture provides a global decomposition representation across specific frequency bands. Low‐frequency components primarily contain noise, typically manifesting as repetitive, textureless regions. Conversely, high‐frequency components represent target regions rich in texture.

**FIGURE 3:**
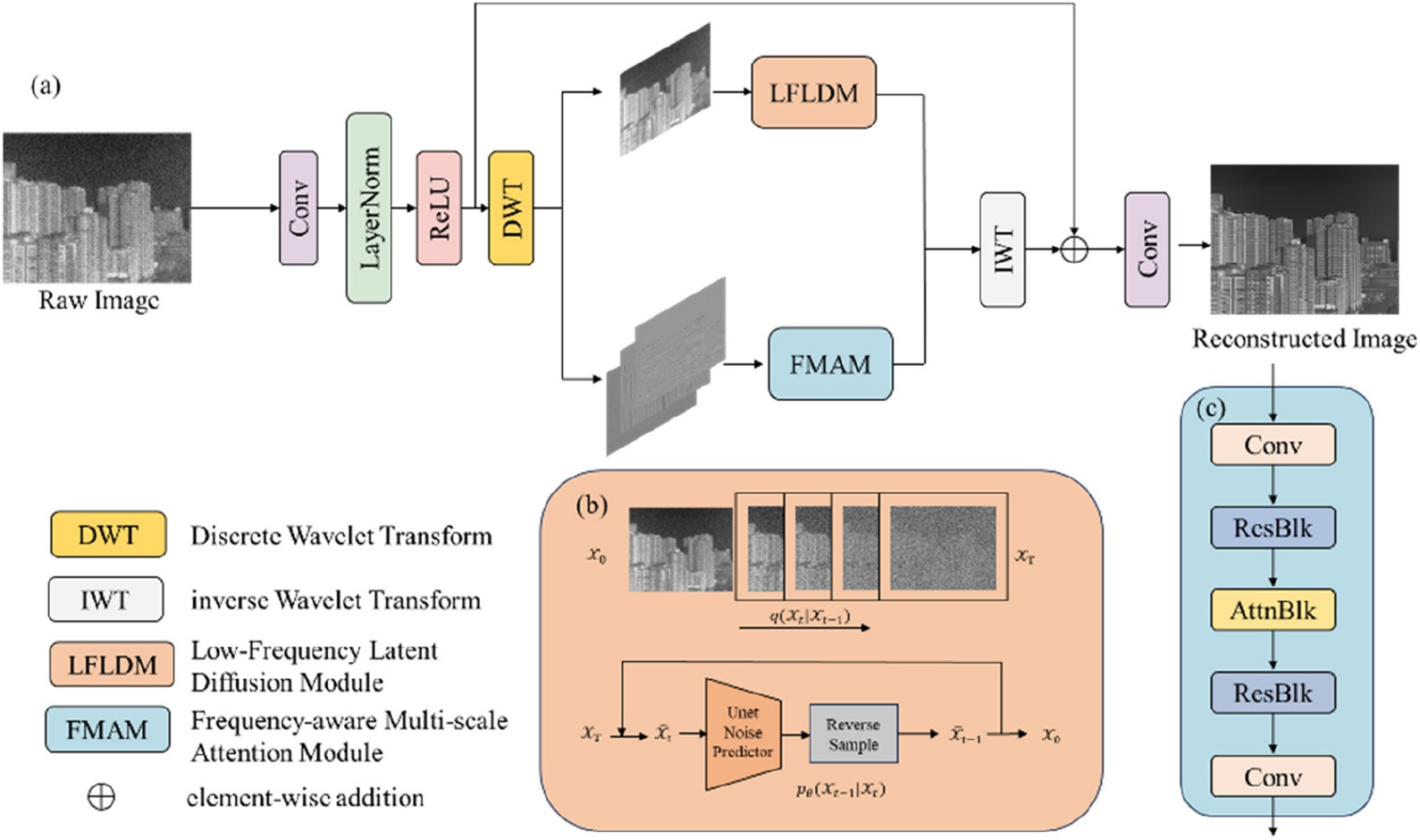
Architectural Overview of the IFEDiff‐Net framework. (a) This framework takes blurred infrared images as input and outputs clear images. We employ wavelet transforms to separate high‐frequency and low‐frequency information, processing each component through dedicated modules. (b) The low‐frequency latent diffusion structure is shown in panel (a). The diffusion process preserves global features during progressive denoising while removing redundant noise in low‐frequency regions. (c) The implementation of the frequency‐aware multi‐scale attention module shown in panel (a) dynamically adjusts weights across different frequency bands.

Discrete wavelet transform (DWT) is employed to decompose the input image features into low‐frequency and multi‐scale high‐frequency sub‐bands. As shown in Figure 3b, a low‐frequency latent diffusion module (LFLDM) is introduced to perform iterative denoising on the low‐frequency subband, effectively preserving the global structural information. For the high‐frequency subbands, as shown in Figure 3c, a Frequency‐aware multi‐scale attention module (FMAM) is designed to adaptively enhance textures and edges through global attention, alleviating high‐frequency detail degradation. A deep encoding module composed of alternating residual and attention blocks is used to refine feature representations. The processed subband features are fused via inverse wavelet transform (IWT), enabling image reconstruction with improved structural consistency and detail clarity.

We designed a composite loss function *L* to optimize the image reconstruction process. This loss function integrates both the pixel‐level reconstruction loss *L*_rec_ and the frequency‐domain consistency loss *L*_spec_. The pixel‐level reconstruction loss *L*_rec_ employs the *L*_1_ norm, minimizing pixel‐level errors between the degraded image *I*_d_ and the true image *I*_g_ to ensure the reconstructed image visually approximates the true image. *L*_rec_ is calculated as follows:

(4) $$L_{\text{rec}}=\frac{1}{N}\sum_{i=1}^{N}{\left\|I_{d}\left(i\right)-I_{g}\left(i\right)\right\|}_{1},$$

where ${\left\|\cdot \right\|}_{1}$ denotes the *L*_1_ norm, representing the sum of absolute errors. This loss function exhibits strong robustness against outliers and better preserves image edges and texture details. The frequency‐domain consistency loss *L*_spec_ employs the *L*_2_ norm to measure the similarity between the degraded image *I*_d_ and the true image *I*_g_ in the frequency domain, as follows:

(5) $$L_{\text{spec}}=\frac{1}{M}\sum_{j=1}^{M}{\left\|F\left(I_{d}\right)\left(j\right)-F\left(I_{g}\right)\left(j\right)\right\|}_{2}^{2},$$

where F(·) denotes the Fourier transform operation. We can directly measure the energy difference between signals in the frequency domain, thereby optimizing the global consistency of images, particularly excelling in the recovery of high‐frequency components.

To balance pixel‐level and frequency‐domain losses, we introduce two weighting coefficients *α* and *β*. The composite loss function *L* is defined as follows:

(6) $$L=\alpha L_{\text{rec}}+\beta L_{\text{spec}}.$$

### 2.4 Experimental Results

The optimized design parameters were implemented through UV‐assisted nanoimprint lithography, where nanoscale patterns were replicated from a mold onto the substrate and then cured to form the designed metalens structure [36]. The fabricated metalens is shown in Figure 4a. The surface morphology was examined by scanning electron microscopy as shown in Figure 4b. The nanocolumn structures are consistent with the design geometry. The complete lens assembly weighs 94 g. The assembled prototype is shown in Figure 4c. The mechanical assembly of the lens is illustrated in Supporting Information S1: Figure S3a. The housing is fabricated from aluminum alloy 6061 and treated with black anodization to enhance surface durability and suppress stray reflections. The lens is securely mounted to the L615 long‐wave cooled infrared core via an M59 × 0.75 mm internal threaded interface, as shown in Supporting Information S1: Figure S3b. After engagement with the internal thread of the cooled detector module, the portion of the lens extending beyond the detector measures 26.76 mm in length. High‐pass and low‐pass filters were incorporated at the system exit pupil to minimize the influence of wavelengths outside the design range, improving imaging quality. An L615 cooled infrared detector with a pixel size of 15 μm (640 × 512 resolution) was used for validation. The cutoff frequency for the detector's imaging is determined by the pixel size. It is approximately 33.33 lp/mm. The detector's noise equivalent temperature difference (NETD) is less than 25 mK at 25°C. The detector ensures minimal thermal noise interference, enhancing the quality of the captured thermal images.

**FIGURE 4:**
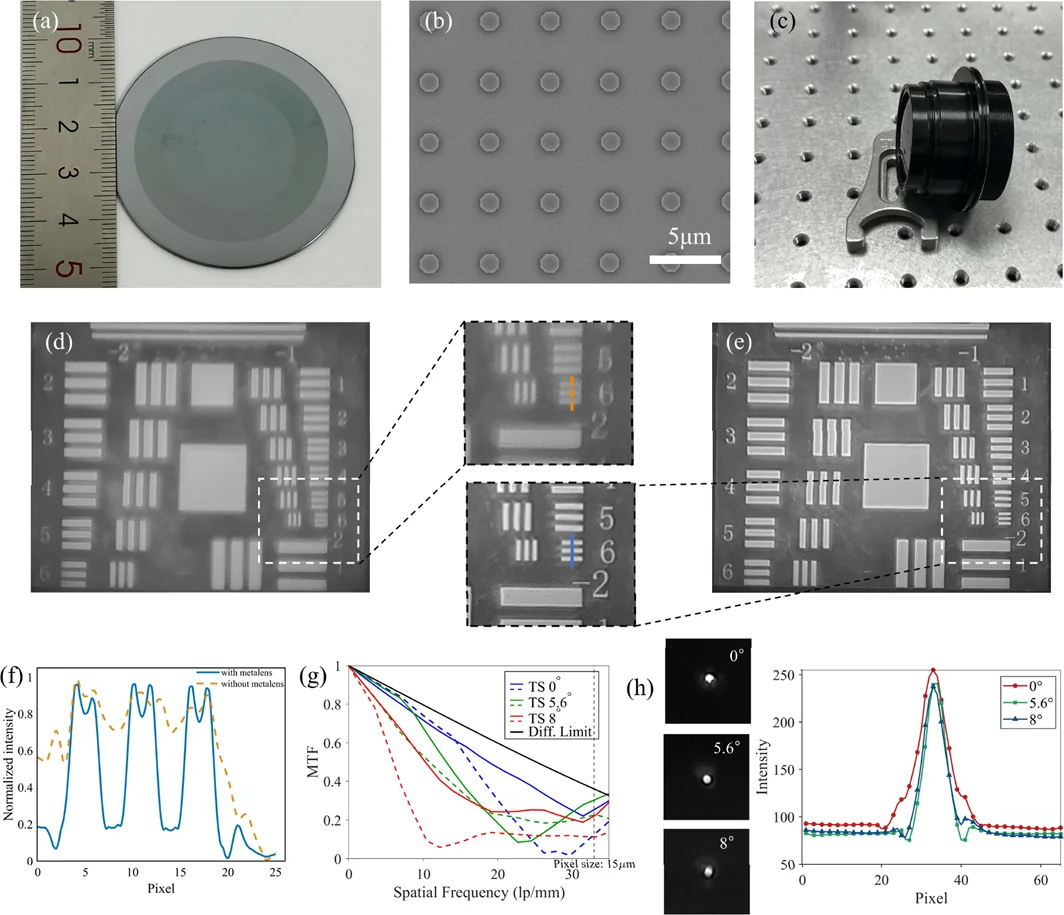
Experimental characterization of the hybrid refractive–metalens imaging system. (a) Photograph of the fabricated all‐germanium metalens. (b) Scanning electron microscopy image of the metalens nanostructures. (c) Photograph of the assembled LWIR hybrid refractive metalens imaging system. (d, e) Thermal images of the resolution target captured by the single refractive lens system and the hybrid refractive metalens system, respectively. The enlarged regions show the selected horizontal and vertical line pair features of Group 6 at the −1 target region. (f) Normalized intensity profiles extracted from the selected line pair regions in (d) and (e), showing the improved contrast after introducing the metalens. (g) Experimental MTF curves measured at different field angles. (h) Measured PSFs at different field angles and the corresponding intensity profiles. The point target was generated by imaging a blackbody source through a circular aperture and a collimator.

Thermal imaging experiments were performed using an aluminum resolution target, as shown in Figure 4d,e. The target was heated to 50°C and imaged at a distance of 3.7 m. Imaging experiments were conducted using both the single refractive lens system and the hybrid refractive metalens system. Compared with the single refractive lens system, the hybrid system resolves the Group‐6 line‐pair features more clearly in both horizontal and vertical directions, with sharper edge transitions and higher contrast. The enlarged regions in Figure 4d,e further confirm the improved resolution after introducing the metalens. The corresponding normalized intensity profiles are shown in Figure 4f, where the hybrid system exhibits larger modulation depth than the single refractive lens system. As shown in Figure 4g, the measured MTF remains higher than 0.2 near the detector Nyquist frequency, consistent with the optical design. The measured PSFs at different field angles are shown in Figure 4h. The PSF profiles remain compact and show limited variation across different fields of view, indicating that the assembled hybrid system effectively suppresses aberrations. To further enhance imaging quality, imaging experiments were conducted on outdoor scenes, followed by image restoration using neural networks. Several representative infrared enhancement algorithms were trained and compared. The restoration results show that the proposed algorithm achieves the best reconstruction performance among the compared methods.

The training dataset was obtained from the high resolution dataset for Raytrontek [37], covering multiple diverse scenarios. Both training and testing were implemented using PyTorch on an NVIDIA GeForce RTX 4090 GPU. During training, we employed a stochastic gradient descent (SGD) optimizer with an initial learning rate of 10^−3^ for 10,000 iterations, using an initial batch size of 16. To adaptively adjust the learning rate, a stepwise decay strategy was adopted, reducing the learning rate by one order of magnitude (i.e., multiplying by 0.1) every 3000 iterations. The weight values *α* and *β* of the loss function were set to 0.1 and 0.3, respectively. This gradual reduction enabled stable convergence during optimization while preventing overfitting.

We evaluate restoration performance on a simulated dataset containing 500 test images. Peak signal‐to‐noise ratio [38] (PSNR) and structural similarity index measure [39] (SSIM) quantify reconstruction accuracy with respect to the ground truth. We further collect 50 real‐world images from diverse scenes for experimental validation. As no reference data are available, restoration quality is assessed using the blind/referenceless image spatial quality evaluator [40] (BRISQUE), contrast enhancement‐based image quality [41] (CEIQ), naturalness image quality evaluator [42] (NIQE), and perception‐based image quality evaluator [43] (PIQE), as summarized in Table 1. These metrics characterize image quality in terms of naturalness, perceptual fidelity, and detail preservation by analyzing local statistics, contrast, texture complexity, and visual consistency.

**TABLE 1:** Comparison of quantitative results for reconstructed images from synthetic and real‐world infrared datasets.

Methods	Synthetic dataset		Real‐world infrared dataset			
	SSIM↑	PSNR↑	BRISQUE↓	CEIQ↑	NIQE↓	PIQE↓
Input	0.75	22.47	46.29	2.78	5.23	78.09
DSDNet [44]	0.83	24.81	42.23	2.72	4.31	45.69
DeblurGAN‐v2 [45]	0.89	25.43	32.15	2.84	4.59	**33.04**
MPRNet [46]	0.81	24.21	44.13	2.79	4.96	58.97
MLWNet [47]	0.89	25.08	40.93	2.83	4.36	43.22
MIMO‐UNet [48]	0.79	23.18	43.77	2.87	5.28	64.32
NAFNet [49]	0.86	24.94	39.4	2.89	4.18	41.85
Ours	**0.92**	**26.18**	**29.83**	**2.91**	**4.11**	39.20

*Note:* Bold text denotes the best results.

The restoration performance was evaluated on both simulated infrared images and real‐world images captured by the hybrid refractive–metalens system. The simulation‐based reconstruction results and comparisons with representative restoration algorithms are presented in Supporting Information S1: Figure S4. For real‐world infrared images, Figure 5 shows representative directly captured blurry images and the corresponding results restored by the proposed IFEDiff‐Net, whereas the complete qualitative comparison with other algorithms is provided in Supporting Information S1: Figure S5. The restored images preserve sharper structural boundaries and suppress residual blur, improving the visibility of building edges, vehicle contours, and human silhouettes. As summarized in Table 1, IFEDiff‐Net improves SSIM and PSNR by 22.7% and 16.5%, respectively, over the degraded input on the synthetic dataset, and by 3.4% and 3.0%, respectively, over the strongest baseline results. On real‐world infrared images, IFEDiff‐Net reduces BRISQUE and NIQE by 35.6% and 21.4%, respectively, while improving CEIQ by 4.7% compared with the input images. Ablation experiments of the network modules and loss functions are detailed in Supporting Information S1: Note S6 and summarized in Supporting Information S1: Tables S2 and S3, respectively. Noise‐level evaluation was further performed under the original and progressively increased Gaussian and impulse noise settings, and the corresponding PSNR and SSIM results are reported in Supporting Information S1: Table S4. The performance degradation observed in these variants confirms the complementary roles of low‐frequency denoising, high‐frequency detail enhancement, and frequency‐domain supervision. These results indicate that the proposed frequency‐aware restoration network effectively compensates for residual degradation in the hybrid refractive–metalens imaging system and improves the visual quality of real infrared images.

**FIGURE 5:**
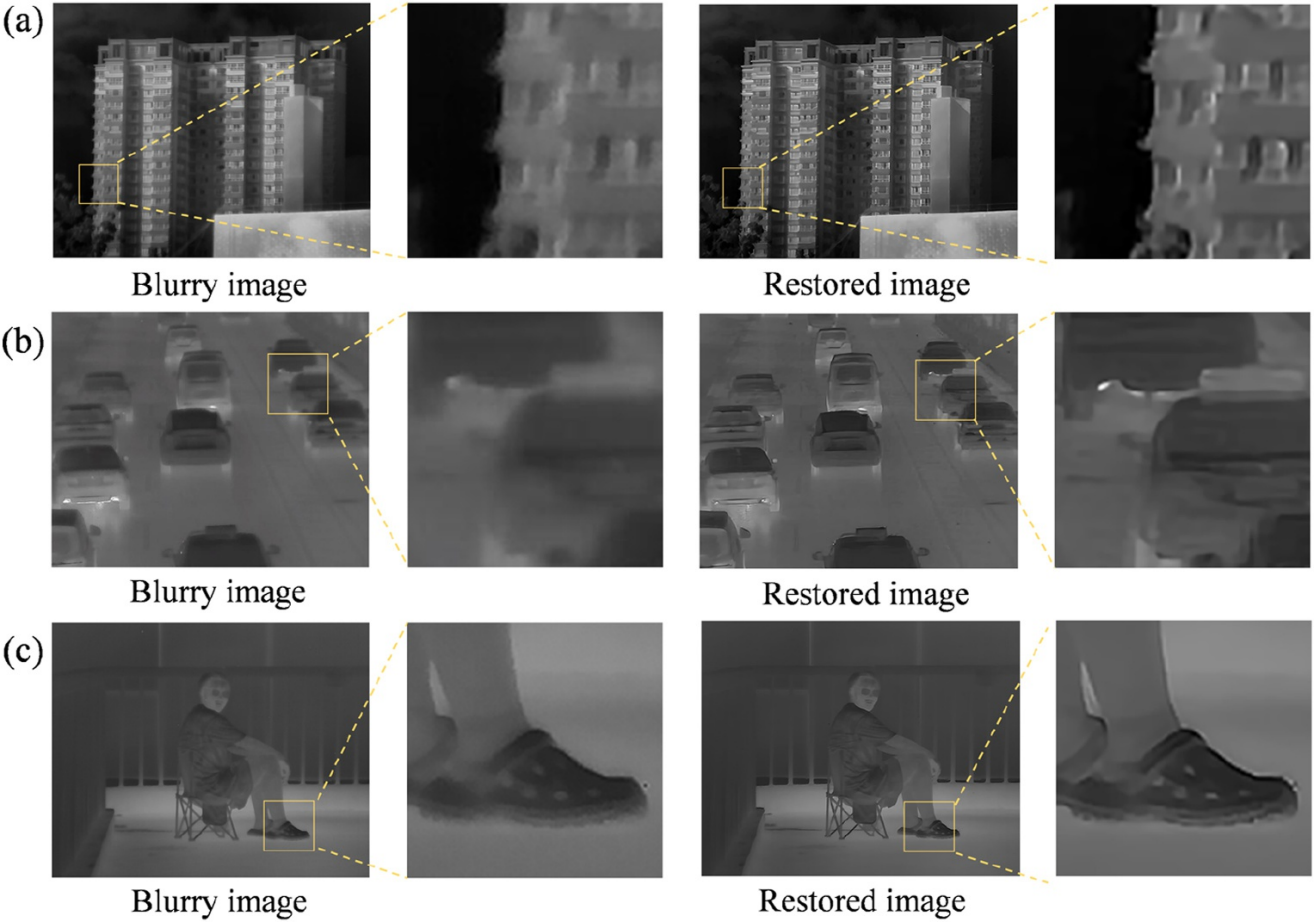
Real‐world infrared image restoration results. (a–c) Three representative scenes captured by the hybrid refractive metalens system. Each row shows the directly captured blurry image and the restored result with zoomed‐in views. The individuals depicted in the figures have provided consent for publication of images.

## 3 Conclusions

In this paper, we demonstrated a large‐aperture hybrid refractive–metalens imaging system for LWIR applications, with a clear aperture of 40 mm and operation over the 7.7–9.5 μm band. A comparison with representative hybrid refractive metalens systems is summarized in Supporting Information S1: Table S5. The optical optimization relied on an integrated model of the complete hybrid system. This formulation unified the evaluation of geometric aberrations and wave optical effects without a full‐wave model of the entire aperture. The fabricated all‐germanium prototype has a total lens weight of 94 g and an axial length extending beyond the detector of 26.76 mm, while achieving a measured MTF higher than 0.2 at the detector Nyquist frequency. To further improve the captured infrared images, a frequency‐aware neural reconstruction framework was employed to compensate for residual blur, detector noise, and image degradation caused by residual aberrations and system imperfections. Computational restoration mitigates residual image degradation, achieving a 16.5% improvement in PSNR for full‐reference synthetic evaluation and a 35.6% reduction in BRISQUE for reference‐free real‐world infrared evaluation. The results demonstrate the practical feasibility of scaling hybrid refractive–metalens optics toward compact, lightweight, and computationally enhanced LWIR imaging systems.

## Author Contributions

**Yinpeng Ma:** conceptualization, methodology, software, visualization, writing – original draft. **Huachao Cheng:** investigation, validation. **Shijie Wei:** methodology, software. **Ben Xue:** data curation, validation. **Xihang Yang:** data curation, formal analysis. **Tengfei Wu:** validation, writing – review and editing. **Yue Wang:** software, validation. **Ruixiang Yang:** software, validation. **Qingqing Guo:** visualization, investigation. **Teli Xi:** funding acquisition, writing – review and editing, project administration, supervision.

## Funding

This work was supported by the National Natural Science Foundation of China (No. 12404387), the Shaanxi Provincial Key Research and Development Program (No. 2024CY‐JJQ‐61), and the Fundamental Research Funds for the Central Universities (No. 20101257867).

## Conflicts of Interest

The authors declare no conflicts of interest.

## Supporting information


Supporting Information S1

## Data Availability

The data that support the findings of this work are available from the corresponding author upon reasonable request.
