# Supplementary material for: Design of Large‐Aperture Hybrid Refractive Metalens System
Source: Nanophotonics. 2026 Sep 18;15(18):e70301. doi: 10.1002/nap2.70301 (PMC13672123; doi:10.1002/nap2.70301)
Supplement: Supplementary file 1 [file NAP2-15-e70301-s001.docx]

Supporting Information

**Design of Large-Aperture Hybrid Refractive Metalens System**

Yinpeng Ma1, Huachao Cheng1, Shijie Wei2, Ben Xue1, Xihang Yang1,2, Tengfei Wu2, Yue Wang1, Ruixiang Yang1, Qingqing Guo1, Teli Xi1*

**Supplementary Note 1: Optimized wavefront results of the hybrid metalens.**

**Figure S1.** Comparison of wavefront distributions between the single refractive lens and the optimized hybrid metalens.

The first-level optimization was performed using a forward wavefront-constrained genetic algorithm for the hybrid refractive metalens system. The optimized variables included the curvature *c*, lens thickness *d*, conic constant *k*, and aspheric coefficients *A*_2_, *A*_4_, and *A*_6_ of the Ge refractive lens, the axial spacing *z_1_* between the refractive lens and the metalens, and the continuous phase distribution *ϕ_MS_*(*x_1_*,*y_1_*;*λ*) of the metalens. The aperture was fixed at D=40 mm as a system specification rather than treated as an optimization variable. The search space was constrained by optical and fabrication requirements, including the lens thickness range, conic-constant range, maximum surface sag variation, local surface slope, phase-gradient variation, and image-plane response.

An initial population of candidate solutions was randomly generated within the predefined constraints. For each candidate, ray tracing was first performed to calculate the optical path length and the wavefront transmitted by the Ge aspheric lens. The transmitted complex field was then propagated through the metalens and to the image plane using the angular spectrum method. The RMS wavefront error averaged over the sampled wavelengths and field angles was used as the primary fitness metric, together with penalty terms for excessive surface sag, large phase-gradient variation, and insufficient MTF at the target spatial frequency. The genetic algorithm updated the candidate population through selection, crossover, and mutation to minimize the fitness value. The optimization was terminated when the fitness value reached a stable minimum or when the maximum number of generations was reached. The optimized system-level parameters were then used to generate the continuous metalens phase for subsequent unit-cell mapping.

**Table S1.** PV and RMS wavefront aberrations of the single refractive lens compared with the hybrid metalens.

|  | single refractive lens | hybrid metalens |
| --- | --- | --- |
| RMS | 0.6927 | 0.3853 |
| PV | 2.8314 | 1.7802 |

**Supplementary Note 2: Imaging and noise simulation of cooled infrared detectors.**

**Figure S2.** Imaging simulation results with spatially varying PSF degradation at different field‑of‑view positions. (a) clear image, (b) optically degraded image, (c) noise-degraded image.

In our experiments, an L615 cooled infrared detector was used to acquire the imaging data. To make the synthetic degradation closer to the real imaging process, noise was added after the spatially variant PSF degradation in the normalized intensity domain. The additive Gaussian noise was used to describe random intensity fluctuations caused by detector readout, background radiation variation, and observation-condition changes. Its standard deviation was set to σ = 0.05, corresponding to a moderate noise level with an equivalent SNR of approximately 26.02 dB. This setting introduces visible noise while preserving the main optical blur characteristics. Sparse impulse noise was further introduced to simulate isolated abnormal pixels caused by defective pixels, dead pixels, transient circuit instability, and environmental interference. The impulse noise probability was set to p = 0.5% per pixel, and the selected pixels were randomly replaced by extreme bright or dark values. The final synthetic degraded images include both the optical degradation from the hybrid refractive metalens system and the mixed noise perturbations related to detector response and practical observation conditions.

**Supplementary Note 3:** **Hybrid refractive metalens was assembled and integrated with cooled infrared detector.**

**Figure S3.** Assembly and integration of the hybrid refractive metalens system. (a) Photograph of the assembled hybrid lens module. (b) Photograph of the hybrid lens integrated with the cooled infrared detector.

**Supplementary Note 4:** **Neural network algorithm simulation dataset restoration results.**

**Figure S4.** Image restoration results on the simulated infrared dataset. Representative synthetic infrared scenes and zoomed-in regions are shown for detailed inspection. The restoration results include the degraded input, DSDNet^1^, DeblurGAN-v2^2^, MPRNet^3^, MLWNet^4^, MIMO-UNet^5^, NAFNet^6^, and the proposed IFEDiff-Net.

**Supplementary Note 5: Real-world infrared image restoration results of the hybrid refractive–metalens system**

**Figure S5.** Real-world infrared image restoration results. Three representative infrared scenes captured by the hybrid refractive metalens system are shown with zoomed-in regions for detailed inspection. The results include the directly captured blurry images, representative methods, and the proposed method.

**Supplementary Note 6:** **Ablation experiments and noise robustness analysis**

To further evaluate the contribution of the proposed frequency-domain processing strategy in IFEDiff-Net, we conducted ablation experiments on both the network modules and the training losses. All ablation models were trained and tested under the same experimental settings, including the same training data, optimizer, learning rate schedule, input resolution, and evaluation protocol. The complete model is denoted as A0. To analyze the effectiveness of each module, we constructed three degraded variants. In A1, the Low-Frequency Latent Diffusion Module was removed, and the low-frequency subband was directly fed into the following fusion stage. In A2, the Frequency-aware Multi-scale Attention Module was removed, so that the high-frequency subbands were no longer enhanced by adaptive multi-scale frequency attention. In A3, both the low-frequency diffusion module and the high-frequency attention module were removed, leaving only the basic wavelet decomposition and reconstruction pathway. This setting allows us to separately examine the contribution of low-frequency denoising, high-frequency detail enhancement, and their joint effect on infrared image restoration.

**Table S2**. Ablation results of the proposed network modules. The best results are highlighted in bold.

| Variant | LFLDM | FMAM | PSNR | SSIM |
| --- | --- | --- | --- | --- |
| A1 | × | √ | 23.53 | 0.86 |
| A2 | √ | × | 21.89 | 0.81 |
| A3 | × | × | 18.67 | 0.72 |
| A0 | √ | √ | 25.94 | 0.93 |

As shown in Table S2, removing either the low-frequency or high-frequency processing module leads to performance degradation. When the Low-Frequency Latent Diffusion Module is removed, the model becomes less effective in suppressing redundant noise and smooth-region artifacts, resulting in lower reconstruction accuracy and weaker perceptual quality. When the Frequency-aware Multi-scale Attention Module is removed, the recovery of edges and fine textures is weakened, especially in regions containing building boundaries, vehicle contours, and human silhouettes. The performance further decreases when both modules are removed, indicating that a simple wavelet decomposition and reconstruction process is insufficient to fully compensate for the residual blur and frequency information loss introduced by the infrared optical imaging system. In contrast, the full model achieves the best overall performance, demonstrating that low-frequency diffusion-based denoising and high-frequency attention-based enhancement play complementary roles in improving infrared image restoration quality.

To further verify the effectiveness of the proposed loss function, we performed another group of ablation experiments by changing the training objective while keeping the complete network architecture unchanged. The complete loss setting is denoted as B0, where both the pixel-level reconstruction loss and the frequency-domain consistency loss are used. In B1, the frequency-domain consistency loss was removed, and the model was optimized only with the pixel-level reconstruction loss. In B2, the pixel-level reconstruction loss was removed, and the model was trained only with the frequency-domain consistency loss. This setting is used to evaluate whether the spatial-domain and frequency-domain constraints provide complementary supervision during infrared image reconstruction.

**Table S3.** Ablation results of the proposed loss functions. The best results are highlighted in bold.

| Variant | *L_rec_* | *L*_spec_ | PSNR | SSIM |
| --- | --- | --- | --- | --- |
| B1 | √ | × | 23.16 | 0.87 |
| B2 | × | √ | 23.53 | 0.89 |
| B0 | √ | √ | 25.71 | 0.93 |

As shown in Table S3, using only the pixel-level reconstruction loss can preserve the overall image structure, but the restored images tend to suffer from over-smoothed edges and insufficient high-frequency details. This indicates that pixel-domain supervision mainly reduces local reconstruction errors but cannot fully constrain the spectral distribution of restored infrared images. In contrast, using only the frequency-domain consistency loss improves part of the spectral response, but the lack of direct spatial-domain supervision may lead to unstable local structures and intensity deviations. The complete loss setting achieves the best performance across the evaluation metrics, suggesting that the pixel-level reconstruction loss and the frequency-domain consistency loss are mutually beneficial. The former ensures spatial fidelity and structural consistency, while the latter further improves frequency distribution recovery and high-frequency detail preservation. These results verify the effectiveness of the proposed frequency-aware optimization strategy for infrared image restoration.

We evaluate the robustness of IFEDiff-Net under varying environmental noise conditions by progressively increasing the noise intensity on top of the original imaging degradation model. During evaluation, the optical degradation component, including the spatially variant PSF, remains unchanged, while only the noise level introduced during image formation is systematically increased to simulate more challenging observation conditions. No retraining, fine-tuning, or test-time adaptation is performed. Following the degradation model described in Supplementary Note 2, two representative environmental noise components are considered: additive Gaussian observation noise and sparse impulse noise. For Gaussian-noise evaluation, the impulse noise component is kept unchanged while the Gaussian noise variance is gradually increased to simulate progressively degraded signal-to-noise conditions. For impulse-noise evaluation, the Gaussian noise level is fixed while the impulse noise probability is progressively increased to model increasing environmental corruption. PSNR and SSIM are computed under each noise level to quantify reconstruction performance across different degradation severities. The results reported in Table S4 show that IFEDiff-Net maintains stable performance under increasing environmental noise levels, demonstrating strong robustness to observation-condition variations and reliable generalization under degraded imaging scenarios.

**Table S4.** Test results of IFEDiff-Net under different noise settings.

| Noise Parameters | SNR (dB) | PSNR↑ | SSIM↑ |
| --- | --- | --- | --- |
| σ = 0.05, p = 0.05% | 16.49 | 26.18 | 0.92 |
| σ = 0.1, p = 0.1% | 10.91 | 23.24 | 0.89 |
| σ = 0.25, p = 0.5% | 7.76 | 18.71 | 0.81 |

**Supplementary Note 7: Comparison with other works**

**Table S5.** Summary of representative hybrid refractive metalens systems, including their diameters, bandwidths, F-numbers, FOVs, and materials.

| Year | Diameter  (mm) | Bandwidth  (μm) | F/# | FOV (°) | Material |
| --- | --- | --- | --- | --- | --- |
| 2018^7^ | 4 | 0.46-0.7 | 6.64 | N/A | TiO₂ |
| 2024^8^ | 6.3 | 8-12 | 1.5 | 20° | Si |
| 2024^9^ | 3.2 | 0.505-0.56 | 0.96 | 30° | Si₃N₄ |
| 2024^10^ | 6 | 8-14 | 0.97 | 30° | Si/ZnS |
| 2025^11^ | 25.4 | 9.2-11.8 | 1.8 | N/A | Si |
| This work | 40 | 7.7-9.5 | 2 | 8° | Ge |

**References**

(1) Kuo, P.-H.; Pan, J.; Chien, S.-Y.; Yang, M.-H. Efficient Non-Blind Image Deblurring with Discriminative Shrinkage Deep Networks. *IEEE Transactions on Circuits and Systems for Video Technology* **2025**. DOI: 10.1109/TCSVT.2025.3553846.

(2) Kupyn, O.; Martyniuk, T.; Wu, J.; Wang, Z. Deblurgan-v2: Deblurring (orders-of-magnitude) faster and better. In *Proceedings of the IEEE/CVF international conference on computer vision*, 2019; pp 8878–8887. DOI: 10.1109/ICCV.2019.00897.

(3) Zamir, S. W.; Arora, A.; Khan, S.; Hayat, M.; Khan, F. S.; Yang, M.-H.; Shao, L. Multi-stage progressive image restoration. In *Proceedings of the IEEE/CVF conference on computer vision and pattern recognition*, 2021; pp 14821–14831. DOI: 10.1109/CVPR46437.2021.01458.

(4) Gao, X.; Qiu, T.; Zhang, X.; Bai, H.; Liu, K.; Huang, X.; Wei, H.; Zhang, G.; Liu, H. Efficient multi-scale network with learnable discrete wavelet transform for blind motion deblurring. In *Proceedings of the IEEE/CVF Conference on Computer Vision and Pattern Recognition*, 2024; pp 2733–2742. DOI: 10.1109/CVPR52733.2024.00264.

(5) Cho, S.-J.; Ji, S.-W.; Hong, J.-P.; Jung, S.-W.; Ko, S.-J. Rethinking coarse-to-fine approach in single image deblurring. In *Proceedings of the IEEE/CVF international conference on computer vision*, 2021; pp 4641–4650. DOI: 10.1109/ICCV48922.2021.00460.

(6) Chen, L.; Chu, X.; Zhang, X.; Sun, J. Simple baselines for image restoration. In *European conference on computer vision*, 2022; Springer: pp 17–33. DOI: 10.1007/978-3-031-20071-7_2.

(7) Chen, W. T.; Zhu, A. Y.; Sisler, J.; Huang, Y. W.; Yousef, K. M. A.; Lee, E.; Qiu, C. W.; Capasso, F. Broadband Achromatic Metasurface-Refractive Optics. *Nano Letters* **2018**, *18* (12), 7801–7808. DOI: 10.1021/acs.nanolett.8b03567.

(8) Liu, M.; Zhao, W.; Wang, Y.; Huo, P.; Zhang, H.; Lu, Y. Q.; Xu, T. Achromatic and Coma-Corrected Hybrid Meta-Optics for High-Performance Thermal Imaging. *Nano Letters* **2024**, *24* (25), 7609–7615. DOI: 10.1021/acs.nanolett.4c01218.

(9) Chen, Q.; Zhou, J.; Pian, S.; Xu, J.; Li, X.; Li, B.; Lu, C.; Wang, Z.; Jiang, Q.; Qin, S.; et al. Hybrid Meta-Optics Enabled Compact Augmented Reality Display with Computational Image Reinforcement. *ACS Photonics* **2024**, *11* (9), 3794–3803. DOI: 10.1021/acsphotonics.4c00989.

(10) Zang, G.; Ren, J.; Shi, Y.; Peng, D.; Zheng, P.; Zheng, K.; Liu, Z.; Wang, Z.; Cheng, X.; Liu, A. Q.; et al. Inverse Design of Aberration-Corrected Hybrid Metalenses for Large Field of View Thermal Imaging Across the Entire Longwave Infrared Atmospheric Window. *ACS Nano* **2024**, *18* (49), 33653–33663. DOI: 10.1021/acsnano.4c12546.

(11) Li, A.; Chen, J.; Liu, M.; Wei, W.; Duan, H.; Jia, H.; Hu, Y. Fundamental design framework of hybrid refractive-metalens system for axial aberrations correction and its validation in LWIR band. *Optics Express* **2025**, *33* (13), 27535–27547. DOI: 10.1364/OE.547921.
